# Transcriptomic dataset of two types of coelomocytes from *Ophelia limacina* (Rathke, 1843)

**DOI:** 10.1016/j.dib.2025.112428

**Published:** 2025-12-31

**Authors:** Daniil Khlebnikov, Polina Belova, Andrey Anisenko

**Affiliations:** a Faculty of Bioengineering and Bioinformatics, Lomonosov Moscow State University, Leninskye Gory 1/73, Moscow 119234, Russian Federation; b Biology Department, Lomonosov Moscow State University, Leninskye Gory 1/12, Moscow, 119234, Russian Federation; c Chemistry Department, Lomonosov Moscow State University, Leninskye Gory 1/10, Moscow, 119234, Russian Federation; d Belozersky Institute of Physico-Chemical Biology, Lomonosov Moscow State University, Leninskie gory 1/40, 119992, Moscow, Russia; e Vavilov Institute of General Genetics of Russian Academy of Sciences, Gubkina 3, 119333 Moscow, Russia

**Keywords:** RNA-seq, Gene expression, Annelids, Coelomocytes

## Abstract

Coelomocytes are a group of specific cells found in the coeloms of invertebrate animals. They perform a variety of functions including immune response, storage, detoxification, and transport of different compounds. *Ophelia limacina*, a member of the phylum Annelida, has two morphologically distinct types of coelomocytes: one of which is amoebocytes, typical for annelids, and the other is rod-bearing cells containing an unusual intracellular structure – a central brown-colored rod. Here we present the transcriptomic data of both cell populations. For this purpose, a total of 148 individuals were collected in the White Sea. The coelomocytes were isolated, both populations of cells (amoebocytes and rod-bearing cells) were enriched using a manual method and then RNA was extracted for NGS-library preparation and further sequencing. Based on obtained data we *de novo* assembled a representative transcriptome of coelomocytes from *O. limacina* using Trinity. Differential expression analysis enabled us to identify transcripts exhibiting significant expression differences between amoebocytes and rod-bearing cells of *O. limacina*.

Specifications Table

**Table:** 

Subject	Biology
Specific subject area	Zoology, transcriptomics
Type of data	Sequence reads obtained after Illumina sequencing of RNA samples, FASTQ files Analyzed data: differential expression analysis, Table, Images, Graph
Data collection	The collection of *Ophelia limacina* was carried out at a depth of 0.5–1 m in June 2024. In total, 148 individuals were collected. The two populations of coelomocytes amoebocytes and rod-bearing cells were enriched and total RNA was extracted from them. This RNA was used for RNA sequencing.
Data source location	The material was collected at sublittoral of Podvolochie Inlet in White Sea near the White Sea Biological Station, Lomonosov Moscow State University 66^о^44’38.3’’N, 33^о^33’32.8’’E.
Data accessibility	The European Nucleotide Archive (ENA), Project: PRJEB102134 [https://www.ebi.ac.uk/ena/browser/view/PRJEB102134] Zenodo [https://doi.org/10.5281/zenodo.16418226]
Related research article	None

## 2 Value of the Data

• These data are important for investigation of invertebrate coelomocyte functions, including immune, nutrient transport and storage, detoxification, etc.
• The representative transcriptome of coelomocytes from *Ophelia limacina* can be used for comparative studies in invertebrate immunology.
• The differential gene expression profiles of amoebocytes and rod-bearing cells obtained can be used for further evaluation of functional differences of these cell types.

## 3 Background

Coelomocytes are a group of cells found in coelomic fluids of different invertebrate animals including annelids. These cells perform a variety of functions among which immune, storage, detoxification and transportation of nutrients, oxygen etc. [1,2]. In different organisms these functions may be combined in one type of coelomocytes or specialization can occur, when a certain function is performed by a certain type of cells. The most studied one is immune function, that involves both direct neutralization of the pathogen through phagocytosis or encapsulation, and indirect action via the secretion of peptides, proteins, and other immunoreactive molecules [1,2].

**Figure 1:**
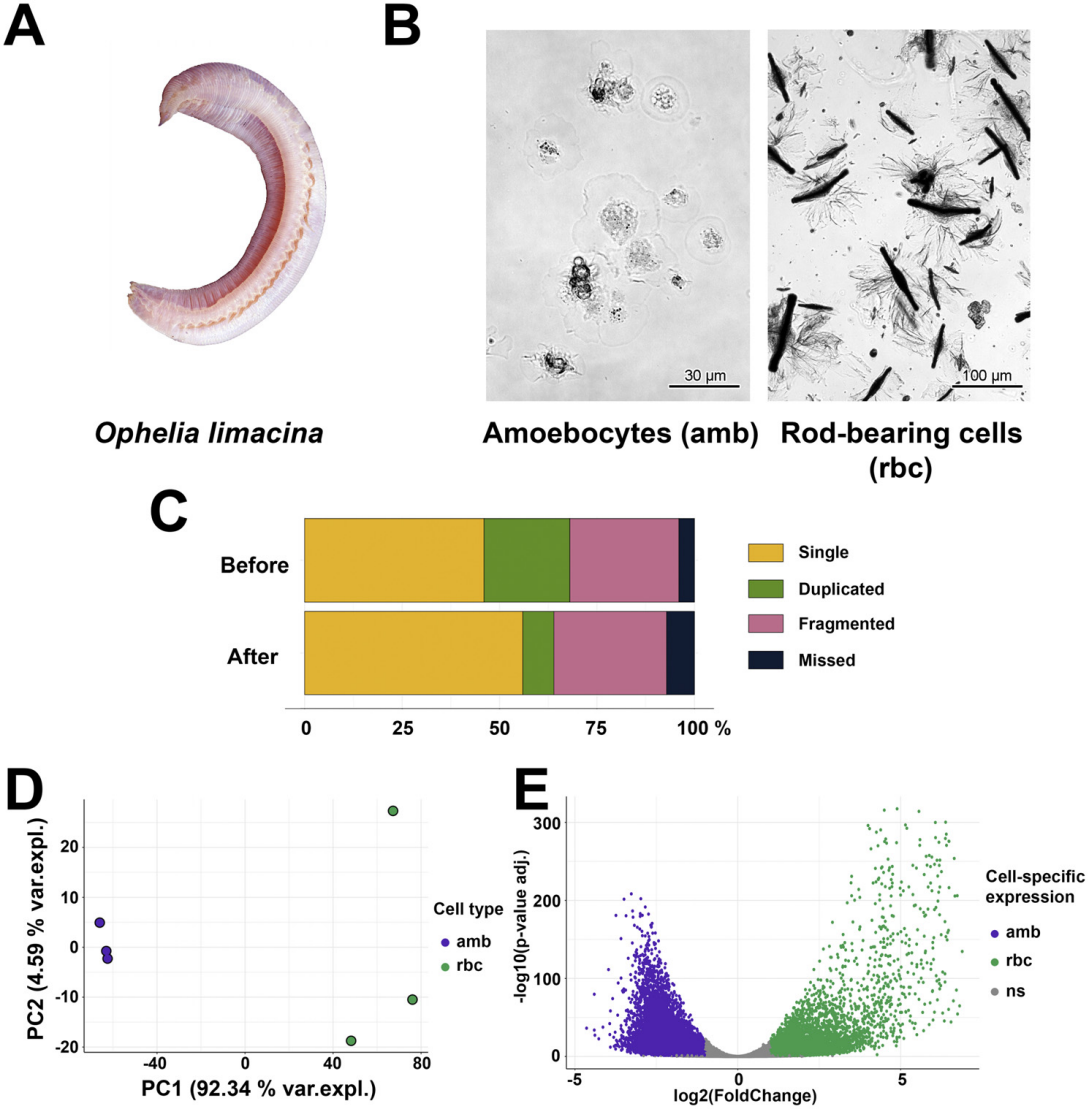
Transcriptomes of two types coelomocytes from *Ophelia limacina*. A – Appearance of *O. limacina*. B – Purity of amoebocytes (amb) and rod-bearing cells (rbc) populations used in study for further transcriptomic characterization. The amb samples contain over 95% of amoebocytes, as assessed by light microscopy. Rod-bearing cells comprised about 75% of the enriched rbc samples. C – BUSCO scores before and after decontamination of assembly. D – Principal component analysis (PCA) of transcriptome-wide normalized gene expression counts assessing the difference in amb and rbc cells. E – Differential transcript expression in amb and rbc cells. Amb-specific transcripts (log2FC < –1 and adj. p-value < 0.05) are highlighted in purple, while rbc-specific transcripts (log2FC > 1 and adj. p-value < 0.05) are displayed in green.

*O. limacina* belonging to Opheliidae family (Fig. 1, A) contains two different types of coelomocytes, one is amoebocytes found in different polychaetes and another one is a rod-bearing cells [3] (Fig. 1, B). The last one contains unique central brown-colored rod and varies greatly in size from 10 to 350 µm. Functions of such type of cells remain unknown [3]. Here we for the first time described the transcriptomic data for two types of *O. limacina* coelomocytes and differentially expressed genes in both subpopulations of them. These data may shed some light on the functions of both types of coelomocytes.

## 4 Data Description

To characterize transcriptomic differences between two types of *Ophelia limacina* coelomocytes –amoebocytes and rod-bearing cells – we developed a manual protocol for separating these populations (described in “EXPERIMENTAL DESIGN, MATERIALS AND METHODS” section). Upon implementation of this protocol, we obtained enriched populations of the cells: amoebocytes with a purity of 95% or greater and rod-bearing cells with about 75% purity assessed by light microscopy (Fig. 1, B). Total RNA from both populations of *O. limacina* coelomocytes was isolated in triplicate. NGS libraries were prepared from the polyadenylated fraction of RNA and sequenced to yield a total of 81.1 Gb reads. Obtained data were deposited at the European Nucleotide Archive (ENA), Project: PRJEB102134.

Trinity software [4] was used to perform *de novo* assembly of representative transcriptome of *O. limacina* coelomocytes. After decontamination and collapse of similar sequences, the final assembly with almost 350-fold coverage was obtained. The decontamination of obtained data led to a reduction of duplicated genes (Fig. 1, C). Decontaminated assembly deposited in Zenodo repository together with counts data doi.org/10.5281/zenodo.16418226.

The final transcriptome of *O. limacina* coelomocytes comprised 447,042 genes and 511,699 transcripts according to Trinity terminology [4]. Among them 29,196 transcripts corresponding to 28,872 genes were identified as protein coding with peptide lengths exceeding 100 amino acids. Notably, 40% of identified peptides had a BLASTP hit against the Uniprot/Swiss-Prot database (e-value threshold of 1e-5) and 36% peptides had at least one domain according to Pfam database. The remaining peptides, constituting 54% of the total number, exhibited no significant sequence homology to known proteins in the databases. Possibly, these proteins are lineage specific.

To quantitatively characterize the expression levels of transcripts in amoebocytes and rod-bearing cells, the reads were mapped to the assembled transcriptome. Additionally, we filtered out transcripts with expression levels below 1 TPM in at least two out of three replicates for each cell population. After processing, transcripts of 48,791 genes remained in the dataset. The total length of transcriptome, calculated by considering only one isoform with the highest expression level per gene in each cell type, was 26.37 Mb.

The cross-sample normalized values (TMM, in transcript per million units) after mapping reads are available at Zenodo repository doi.org/10.5281/zenodo.16418226. Principal component analysis (PCA) following regularized-logarithm transformation demonstrated that the samples exhibited clear clustering within the same cell type and each replicate was suitable for further analysis (Fig. 1, D).

Differential expression analysis was performed using the DESeq2 package [5] by calculating the log2(Fold change) values for transcript abundance and adjusted p-values. The volcano plot illustrates the differentially expressed genes between amoebocytes and rod-bearing cells, highlighting those with absolute log2FC exceeding 1 and adjusted p-value below 0.05 (Fig. 1, E). In amoebocytes, 2,843 up-regulated protein-coding genes were identified, among which 872 (30.67%) had BLAST or Pfam hits. Similarly, in rod-bearing cells, 1,538 up-regulated protein-coding genes were detected, including 795 (51.69%) with BLAST or Pfam hits.

## 5 Experimental Design, Materials and Methods

### 5.1 Collection of material

The 148 adult specimens of *O. limacina* were collected at sublittoral (0.5-1 m depth) of Podvolochie Inlet (66^о^44’38.3’’N, 33^о^33’32.8’’E) in White Sea near the White Sea Biological Station, Lomonosov Moscow State University, in June 2024. The individuals were maintained in a fresh sea water at 10-12°C for 1 week with daily replacement of water to the fresh one until cell isolation.

### 5.2 Coelomocytes isolation and RNA extraction

To isolate coelomocytes, *O. limacina* specimens were placed into 200 µL of 0.22 µm filtered sea water in the 24-well cell culture plate (1 specimen per well). The body wall was dissected with microsurgical scissors (MST, USA) to extract coelomic fluid containing the cells, after which the specimen was removed from the well. Massive contaminations of coelomic fluids with gametes or parasites such as gregarines were verified by light microscopy using Nikon Eclipse TS100 inverted microscope (Nikon, Japan). The 34 samples that did not pass these criteria were further excluded from the analysis. Amoebocyte and rod-bearing cell populations were enriched by manual method based on their different ability in adhesion to the surface of TC-treated plates (Fig. 1, B). Implementation of automatic assays for cell populations separation, e.g. FACS, was impossible due to giant size of rod-bearing cells up to 350 µm [3]. Amoebocytes settled down and attached to the well surface within 10 minutes, after which the coelomic fluid containing rod-bearing cells was collected manually using Pasteur pipete in 1.5 mL tubes under light microscopy control. Amoebocytes were washed with filtered sea water and lysed directly in wells by TRIzol reagent (Invitrogen, USA). The final purity of amoebocyte samples was greater than 95 % for each wells, which was checked using light microscopy. Cell lysates were pooled in three tubes so that each contained lysates from 38 wells. Rod bearing cells from the same wells also were pooled and then centrifuged at 100 g for 5 min, washed once with filtered sea water and lysed by TRIzol reagent (Invitrogen, USA). The content of rod-bearing cells in rbc samples was no less than 75 %. All the steps above were carried out at 4°C.

The RNA extraction from the samples was performed using TRIzol reagent according to the manufacturer’s protocol. To remove genomic DNA from the samples the extracted RNA was treated with DNase I (Thermo, USA) with followed DNase I removal by CleanRNA Standard kit (Evrogen, Russia). The integrity of RNA was analyzed with 1 % agarose gel electrophoresis in TAE buffer with ethidium bromide staining, and on-chip electrophoresis (Bioanalyzer 2100, Agilent). The RIN data are presented in Table 1.

**Table 1:** RNA-seq samples, read metrics, and public ENA sample accessions.

Sample name	Cell type	RIN	Number of raw read pairs (M)	Number of quality trimmed reads (M)	Uniquely mapped reads,%	ENA sample accession
amb1	Amoebocytes	8.3	42.8	42.7	87.2	SAMEA120473375
amb2	Amoebocytes	8.4	47.9	47.9	91.1	SAMEA120473376
amb3	Amoebocytes	8.3	42.2	42.1	84.6	SAMEA120473377
rbc1	Rod-bearing cells	8.4	51.7	51.6	89.8	SAMEA120473378
rbc2	Rod-bearing cells	8.4	40.0	40.0	91.9	SAMEA120473379
rbc3	Rod-bearing cells	8.3	45.9	45.9	96.1	SAMEA120473380

### 5.3 Illumina library preparation and sequencing

The cDNA libraries were prepared from the polyadenylated RNA fraction using TruSeq2 chemistry according to manufacturer’s protocol. The average insert size for all samples was 300-350 bp. Library amplification was carried out for 12 cycles. The final concentration of the libraries ranged from 2.3 to 4.2 ng/µL. The obtained libraries were sequenced on an Illumina NovaSeq6000 instrument with 150 bp long paired-end reads and a yield of at least 40 million reads. Raw reads from the instrument were converted to FASTQ format with bcl2fastq2 software (Illumina). The obtained data are summarized in Table 1.

### 5.4 Transcriptome assembly and annotation

The process of *de novo* transcriptome assembly was carried out utilizing an adapted workflow originally detailed by E. Nikitenko et al. [6]. To ensure complete transparency, we provide a comprehensive overview of the entire methodology employed for processing raw sequencing data. The initial stage involved preprocessing of paired-end reads through a series of quality control steps. Error correction was initially performed using Rcorrector [7] version 1.0.6, targeting k-mer inaccuracies. Subsequently, read pairs containing uncorrectable errors or flagged k-mers were excluded from further analysis. Quality trimming was executed via TrimGalore version 0.6.10, employing specific parameters (“–length 36 -q 5 –stringency 1 -e 0.1”) to filter out sequences of suboptimal quality or insufficient length. Following quality control, reads were subjected to ribosomal RNA filtering using bowtie2 [8] version 2.5.4 against a rRNA database comprising SILVA 138.2 large and small subunit rRNA sequences (LSUParc and SSUParc). Only non-matching reads proceeded to the assembly stage. *De novo* assembly was conducted using Trinity [4] version 2.15.2 with standard parameters. Post-assembly processing included transcript collapsing with cd-hit-est [9] version 4.8.1, applying a 95% identity threshold to remove sequence polymorphisms. Contamination removal was performed using MCSC [10], focusing on Annelida phylum with a clustering parameter n = 4. The resulting clean assembly served as input for transcript quantification analyses. Open reading frame (ORF) prediction was carried out using TransDecoder v5.5.0, retaining sequences exceeding 100 amino acids. Functional annotation was performed through the Trinotate [11] v4.0.2 pipeline, incorporating: protein homology searches via diamond [12] v2.0.15 (BLASTP and BLASTX) against UniProtKB/SwissProt, domain analysis using hmmer [13] v3.3.2, functional annotation through eggNOG [14] v2.1.9 against the Pfam database. This multi-step approach ensured comprehensive processing and accurate annotation of the generated transcriptome dataset.

### 5.5 Identification and analysis of differentially expressed genes

Differential gene expression was conducted utilizing the DESeq2 [15] package after the raw data processing. The workflow comprised several sequential steps. First, the raw reads were mapped to the transcriptome assembly using bowtie2 v2.5.4. Subsequently, contig abundance estimation was performed employing salmon [8] v1.10.3. The resulting data were then processed using DESeq2. Visualization of results was accomplished using the ggplot2 package in R. Transcripts were deemed as differentially expressed if the expression level changed more than 2-fold (Log2FC > 1) between groups and the adjusted p-value was less than 0.05.

### 5.6 Quality control

FastQC was used to assess sequencing quality before and after adapter trimming and filtering for quality and length. The quality of the assembly was checked by mapping the source reads to the assembly as well as by BUSCO [16].

## Limitations

In this study, we employed a manual separation protocol to isolate amoebocyte and rod-bearing cell populations of *Ophelia limacina* coelomocytes for subsequent transcriptomic analysis. The method utilized the distinct adhesion properties of these cell populations to TC-treated plates. Quality assessment demonstrated that the amoebocyte population achieved purity exceeding 95%, while the rod-bearing cell population showed only 75% enrichment. We draw researchers’ attention to the fact that the lower purity level of the rod-bearing cell population may introduce potential biases in the transcriptomic profiles.

## Ethics Statement

The authors have read and follow the ethical requirements for publication in Data in Brief and confirming that the current work does not involve human subjects, animal experiments, or any data collected from social media platforms. *Ophelia limacina* collection did not require specific licensing, however, all the experimental procedures, including material collection, were approved by the Scientific Council of the White Sea Biological Station. All applicable international, national, and/or institutional guidelines for the care and use of animals were followed.

## Credit Author Statement

**Daniil Khlebnikov:** Investigation, Data curation, Methodology, Formal analysis, Software. **Polina Belova:** Investigation, Methodology. **Andrey Anisenko:** Investigation, Validation, Supervision.

## Funding

This work was supported by Interdisciplinary Scientific and Educational Schools of Moscow University No. 23-Ш04-29.

## Data Availability

ENATranscriptomic dataset of two types of coelomocytes from Ophelia limacina (Rathke, 1843) (Original data). ZenodoOphelia limacina transcriptome (Original data).
